# Going Global: Interest in Global Health Among US Otolaryngology Residents

**DOI:** 10.5334/aogh.3283

**Published:** 2021-08-10

**Authors:** Julia Toman, Melynda Barnes Oussayef, J. Zachary Porterfield

**Affiliations:** 1 University of South Florida, Department of Otolaryngology Head and Neck Surgery, Division Facial Plastics and Reconstructive Surgery, University of South Florida, US; 2 Senior Vice President of Medical Affairs and Research – Roman Health Ventures, US; 3 University of Kentucky, Department of Otolaryngology-Head & Neck Surgery, Department of Microbiology, Immunology and Molecular Genetics, Department of Internal Medicine – Division of Infectious Diseases, US; 4 Honorary Senior Lecturer – University of KwaZulu-Natal, School of Clinical Medicine, South Africa

## Abstract

**Background:**

To meet the rising interest in surgical global health, some surgical residency programs offer global health experiences. The level of interest in these programs, however, and their role in residency recruitment and career planning has not been systematically evaluated.

**Objective:**

(1) Define interest in global health among Otolaryngology residents in the USA. (2) Assess engagement of Otolaryngology residencies in global health training. (3) Determine barriers to global health training in residency.

**Methods:**

A survey questionnaire was developed and sent to all Otolaryngology Residency Program Directors for distribution to all current Otolaryngology residents in the US.

**Results:**

A total of 91 complete surveys were collected. A majority of respondents felt that global health was either “very important” or “extremely important” (67%). Two-thirds of respondents had prior global health experience (68%). While 56% of respondents would definitely participate in a global health elective and 78% would likely or definitely participate, only 37% of residency programs offered a global health experience. The availability of a global health elective significantly correlated with residency match choice in respondents with previous global health experience. The three most common barriers to participation were insufficient time, insufficient funding, and lack of program.

**Conclusion:**

Participation in bilateral and equitable international electives is a unique experience of personal and professional growth. There is an interest in these opportunities during residency training among Otolaryngology residents that is not reflected in availability within training programs. This suggests the need for development of humanitarian outreach exposure through global health experiences during surgical residency training.

## Introduction

The values that prompted many to enter the health care profession are closely tied with a core tenet of global health: A passionate desire to serve the underserved [1]. Additionally, global collaboration, which explores sociocultural factors in health care and disparities in its provision around the world, allows for invaluable lessons in patient care, our collective humanity, and teamwork [2]. International experiences during residency training can harness the altruistic desires of young surgeons, reinforce the important historical traditions of the surgeon as a professional dedicated to patients, and encourage volunteerism in all surgeons [3]. Indeed, a more global view of health care provision and need is embraced by an increasing number of residents, including those in surgical fields, highlighting the importance of and role for global health education within residency training [4,5]. For many residents, an awareness of increasing disparities in global health complements a commitment to learning portable skills to serve humanity; for some, this commitment was part of the motivation for a career in surgery [2]. International educational partnerships can provide a unique opportunity to develop resource-use awareness, improved use of history and physical examination skills, a broader knowledge base of surgical pathology, and exposure to key cultural differences in the delivery of healthcare for all parties [2,5,6]. Moreover, recent publications have delineated how these global health experiences touch on all six ACGME core curricula [7,8]. In addition, bilateral, collaborative exchanges can provide opportunities for learning, highlight differences in practice, and encourage novel approaches to solving problems of shared concern.

There is increasing recognition of the role surgery has to play in global health and wellbeing. Inequalities in access to surgical care are stark between high income countries and low- and middle-income countries (LMICs), with ¾ of all surgical procedures globally being performed on only 1/3 of the world’s population [9,10]. However, surgery has often been overlooked in the larger context of global health and global health education [9,10,11]. Global surgery has even been referred to as the neglected step child of global health initiatives [12].

U.S. otolaryngology programs, otolaryngologists and trainees have become increasingly involved in humanitarian efforts [13,14,15]. A few reports exist, examining global health elective development in general surgery and plastic surgery [1,3,5,16,17], including perceived benefit of such a program and the feasibility of program development. However, interest in these programs and their effect on training program selection and career planning has not been evaluated in detail. Moreover, in the otolaryngology literature, evaluation of this topic has been fairly limited but has revealed that an interest in global health exists among trainees [13,15]. The most direct evaluation of this was undertaken by Boyd and Cruz in a focused survey of global health interest among medical students applying to a single residency program. This study found that, among 93% of the 55 respondents, interest in an international rotation was “strong” or “very strong.” This study also ranked the presence of an international rotation among other common deciding factors in residency selection, and found that it ranked behind operative experience, location, lifestyle, research opportunities, and didactics, but ahead of prestige of the program and salary.

As a next step to understanding the benefits of global health experiences and educational partnerships in the field of Otolaryngology, our study expands significantly on the work of Boyd and Cruz in several ways. We obtained responses from US residents who successfully matched into programs across the nation, directly assessed the impact of an international elective on residency selection and career planning, did extensive analysis to determine differences amongst subgroups, and evaluated barriers to participation.

## Methods

A survey questionnaire was developed and underwent review by the Ethics Committee at Yale University. The first portion of the survey collected demographic information such as age and gender, as well as previous international volunteerism. The second portion of the survey sought to elucidate, on a 5-point scale, the level of interest in global health, the role that the presence of a global health program played in residency selection, and future career plans involving global health. Finally, information was collected regarding motivations and barriers to participation in global health volunteerism. Space for free responses was given.

The survey was administered anonymously and voluntarily to all otolaryngology residents in the United States via an email to otolaryngology residency program directors of the 106 ACGME accredited Otolaryngology Residency Program Directors for distribution to their residents. The survey was accessed via a SurveyMonkey© website link in the email. The survey was available for three months, and two reminders for participation were sent via the original method of communication. The estimated total number of residents who could have participated is 1,560.

Responses were tallied and data was compiled for analysis. A Likert-type scale was used to provide ordinal ranks for perceptions of the importance of global health and the likelihood to participate in global health experiences. We utilized standard descriptive statistics, including the Spearman’s rank correlation coefficient, the Mann-Whitney test and Pearson’s chi-squared tests using GraphPad Prism (version 7.0a, www.graphpad.com), which was used for the preparation of figures. A p-value of <0.05 was considered significant for all data endpoints.

## Results

**Figure 1:**
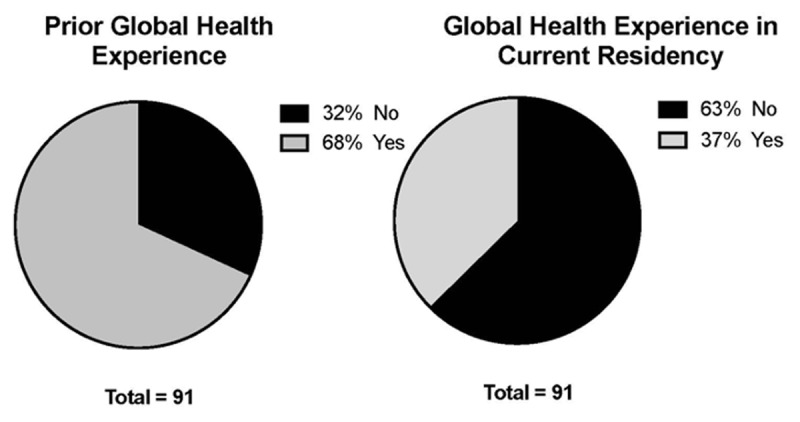
**A**. Prior Global Heath Experience, 68% with prior global health experience. **B**. Global Health Experience Offered in Residency, 37% of programs offer programs.

A total of 91 responses were collected. The average age was 29.8 + 2.8 years; 46% were female and 54% were male. A majority of respondents matched into one of their top three programs (67%, n = 61), with 84% (n = 77) matching into one of their top six programs. There were no significant differences in the level of the match based on gender (p = 0.6277). Sixty-eight percent (n = 62) of respondents had a prior global health experience, yet only 37% (n = 34) of their current residency programs offered a global health or international elective (***Figure 1***).

**Figure 2:**
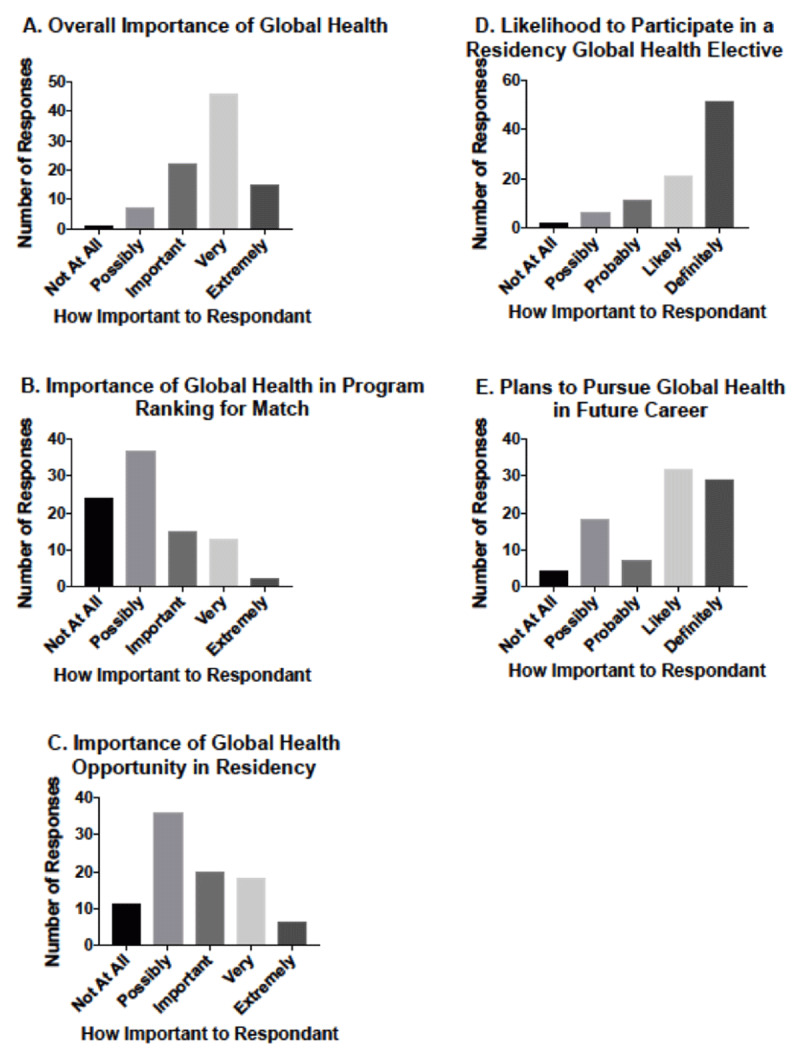
Range of Response to **(A)** Overall Importance of Global Health, **(B)** Importance of Global Health in Match Ranking, **(C)** Importance of having a Global Health Opportunity in Residency, **(D)** The Likelihood of Participating in a Residency Global Health Elective, and **(E)** Plans to Pursue Global Health in Future Career.

A majority of respondents felt that global health was important, with 67% (n = 61) stating it was either “very important” or “extremely important.” However, only 32% (n = 30) felt it was “important,” “very important,” or “extremely important” in their ranking of programs for the National Residency Match Program. A global health opportunity in one’s current residency was felt to be “important,” “very important,” or “extremely important” in 48% (n = 44) of responses (***Figure 2A–C***).

A majority of respondents (56%; n = 51) indicated they would definitely participate in a global health elective during residency, if offered, with only 2% (n = 2) stating they would be not at all likely to participate in such an elective (***Figure 2D***).

A bimodal distribution of respondents was seen with regards to incorporating global health into future career plans, with 67% (n = 61) indicating they would likely or definitely do so and 20% (n = 18) indicating it was only possible they would do so (***Figure 2E***).

**Figure 3:**
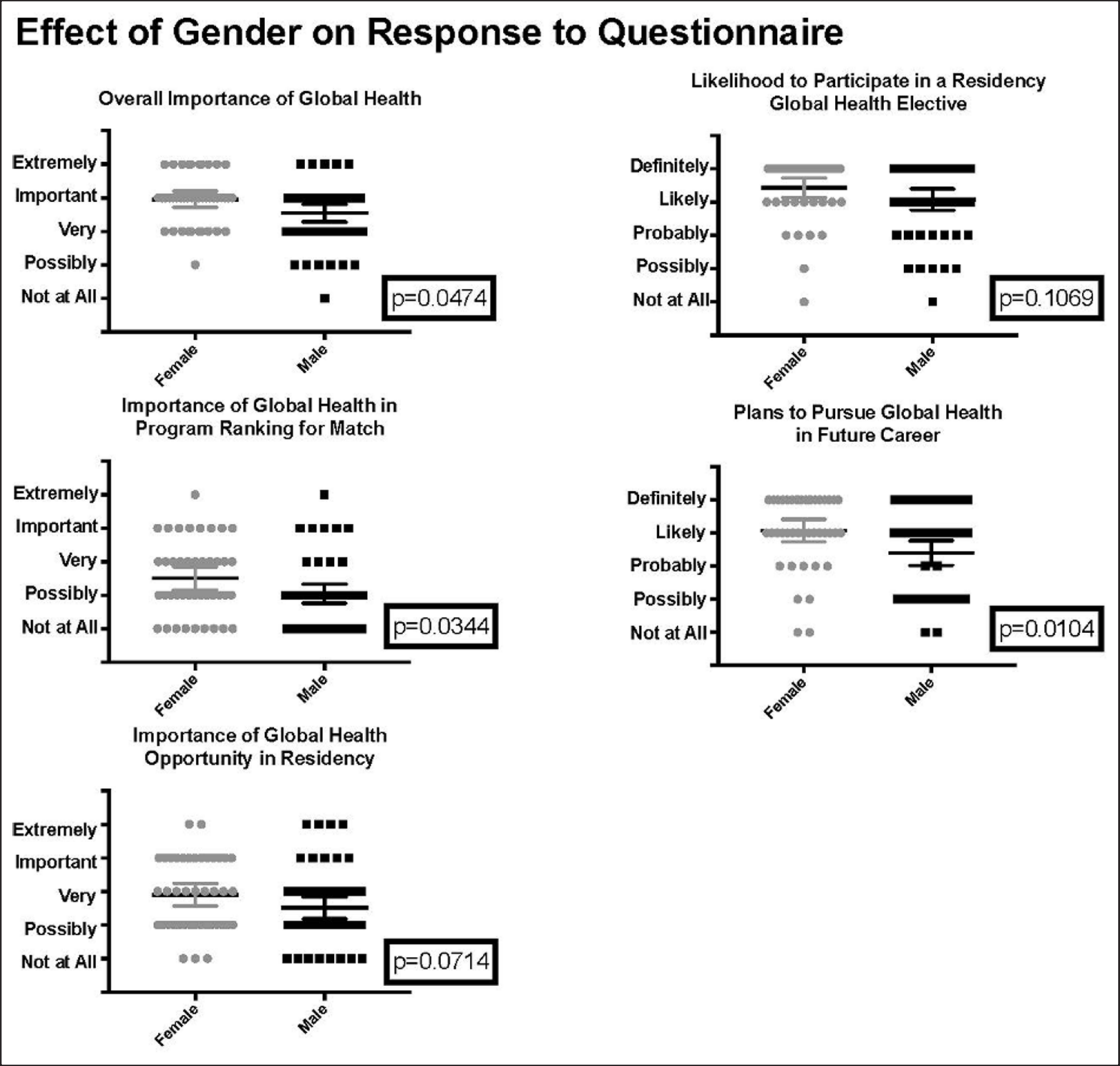
Effect of gender sub-group analysis on response.

Four subgroups were analyzed based on age, gender, level of match, and presence of prior global health experiences. There were no significant correlations between age and responses to questions about the importance of global health or likelihood to participate in a global health experience. Female respondents were more likely to highly rank the overall importance of global health and the importance of global health in their decision regarding their program ranking than their male counterparts (p = 0.0474 and p = 0.0344, respectively) and were more likely to plan to incorporate global health into their future careers (p = 0.0104) (***Figure 3***).

**Figure 4:**
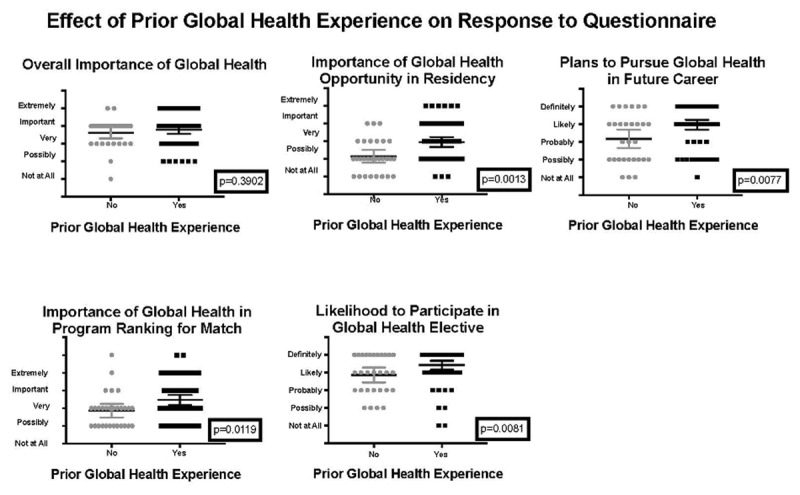
Effect of previous global health experience on survey response.

Prior global health experience had a statistically significant effect on the importance of global health in program ranking for the National Residency Match Program (p = 0.0119), the sense of importance of global health in residency (p = 0.0013), the likelihood to participate in a global health experience during residency if were one to be offered (p = 0.0081), and plans to pursue global health in a future career (p = 0.0077) (***Figure 4***).

The motivations for participating in a global health experience varied. A majority of respondents noted a desire to give back, to travel, to learn about different cultures and to see new and interesting disease pathology; and a feeling that they can make a difference.

The three most common barriers to participation were insufficient time or funding, or a lack of a global health experience.

## Discussion

Our data shows there is a significant interest in global health among otolaryngology residents with a majority indicating that global health was important overall. There is, however, a notable disparity in the number of programs offering a global health experience, with just over 1/3 of programs offering the opportunity, and 79% of respondents who were at least likely to participate in a global health elective.

The corollary to this interest is the role that an interest in global health play in the selection of residency program. While global health education opportunity availability did not significantly affect ranking for the National Residency Match Program for all respondent in our study, there was a statistically significant effect among those individuals with prior global health experiences. This is an expanded nuance from the results from a survey of 2007–2008 Otolaryngology applicants, which did not indicate that the presence of an international elective significantly impacted program choice [14]. The results of this study expand upon the finding of the study conducted by Boyd and Cruz, where 93% of respondents had an interest in international rotations and considered their availability in their residency ranking. A survey by Thompson et al. also showed that availability of an international experience influenced medical student selection of residency program [18].

Our study indicates a growing interest in global health education opportunities. This echoes the results of a survey conducted by Powel et al. in 2007 of surgical trainees, which showed that 58% of respondents would be more interested in a surgery program offering an international experience were they to interview again. The authors concluded that this represents an opportunity to recruit idealistic students who might otherwise be lost to other specialties with a greater global health emphasis [3]. The finding of our study that 80% would participate in an elective if it was offered, indicates an even higher interest. The data indicate that as more programs develop global health experiences, and more trainees with prior global health experience enter residency programs, we can anticipate that the importance of an integrated global health curriculum will continue to grow, and competitive programs may seek to develop and offer these experiences.

Our survey found notable differences based on gender, with female respondents more likely to rate highly the overall importance of global health, the importance of global health in program rankings, the importance of global health in a residency program and plans to incorporate global health into their future career paths. The meaning of this finding remains unclear and requires additional investigation.

Additionally, the data from this study show that prior experience begets interest in future experiences. This is similar to the data from studies conducted in the field of general surgery and a few other surgical sub-specialties. A 2005 survey of UCSF general surgery residents showed that 40% had participated in an international health experience prior to residency, and 90% expressed an interest in an elective in a developing country during residency. A 2009 survey of resident members of the American College of Surgeons found that 92% of the 724 individuals surveyed were interested in international outreach opportunities, and 82% would prioritize these opportunities over other electives [17]. Finally, a study conducted by Aziz et al. of 45 residents who undertook a humanitarian mission during their residency training found that all participants thought that participation in such an activity was a positive part of their training. These missions allowed residents to develop as surgeons and increased cultural competence [16].

The demands of a surgical residency are numerous. Global health education must be balanced against surgical development and case number generation, call responsibilities, and education at the participants’ home institutions. However, global health experiences address all six ACGME core competencies in unique ways [8,19]. The motivations for participation noted by the respondents speak to fulfillment of these competencies with a spirit of volunteerism and education. However, other noted motives include a desire to see new or interesting pathology has the potential for extractionary and colonial global health experiences, however unintended. This speaks to the importance of critical evaluation of global surgical health education opportunities to ensure they are fair and equitable.

Other challenges remain in undertaking international clinical rotations. One of the most common barriers is the availability of opportunities. Volsky and Sinaori conducted a survey of otolaryngology programs’ global health initiatives. They found that one-fourth of programs engaged in global health volunteerism and, of these, 90% supported resident involvement [15]. However, in most postgraduate surgical programs, there are limited opportunities to develop these interests during residency through mentorship, research or clinical experience [2]. Other common difficulties include the need to use vacation time, which was borne out in our study. Other surgical programs such as general surgery and plastic surgery training programs have elective educational time in their curriculum. Allowance of such elective time within Otolaryngology training programs would allow for greater participation for Otolaryngology residents in global health education initiatives. Finally, funding is a constant challenge, with many motivated residents paying out of pocket [3,17]. A study by Hoehn et al. estimated that a two-month surgical rotation could cost over $17,000, which, given the limitations of resident salaries and loan debt burden, will continue to hamper participation [20]. The results of this study highlight financial burden as a persistent barrier to participation and may results in bias in ultimate participation resulting in a disadvantage for less well-off residents.

However, it remains clear that the surgical disciplines, otolaryngology included, continue to lag behind our non-surgical disciplines in developing a global aspect of the educational curriculum [21]. This disparity occurs despite literature showing that international experiences within residency are likely to lead to career development in academic medicine or public health, enhanced proficiency in cross-cultural health care, and graduates’ having an increased commitment to caring for the underserved [18,21]. Additionally, participation in international clinical rotations produces graduates with increased skill and confidence, enhanced sensitivity to cost issues and less reliance on technology [22,23,24,25].

Of notable concern is that most existing global surgical health initiatives did not utilize global health institutional resources or collaborate with international otolaryngology training programs [15]. There is a need for these programs to be undertaken with appropriate level of trainee supervision and in a bilateral, collaborative fashion, in order to continue the process of de-colonization of global health initiatives. While the field has embraced the Taoist mantra “give a man a fish and feed him for a day, teach a man to fish and he will eat for a lifetime,” we in fact propose that the metaphor can be taken a step further to highlight how much we all stand to learn—“fish together and learn from each other.”

### Limitations

There are limitations to these data. We received only 91 responses, which represents just a subset of all of the otolaryngology residents currently in training in the U.S. While it is estimated that the survey could have reached 1,560 residents, the distribution depended upon the forwarding of the survey by the 106 program directors. The relatively low number of responses overall precludes some analyses. Moreover, we may have some degree of voluntary response bias, in which respondents who feel strongly about this issue were more likely to respond, and social desirability bias, in which participants are less likely to respond in a manner that might be deemed socially unacceptable. Additionally, the survey results do not allow for evaluation of the nature of the global health experiences being envisioned. However, these data did provide some key insights into the motivations of otolaryngology residents in program selection and the role that previous global health experience play in both residency selection and future career goals.

## Conclusions

In a rapidly shrinking world, it is increasingly clear that methods need to be established to address health disparities, including the need for surgical care. Given the significant interest in such training opportunities, it is time for surgical training programs, otolaryngology training programs included, to provide training and mentorship for the upcoming generation of surgeons, allowing them to gather the skills they need to participate in this new global environment. There is a need for expansion of bilateral, collaborative surgical global health initiatives that are undertaken with appropriate trainee supervision and with mutual training benefit for both participants.
